# The relationship between long COVID, labor productivity, and socioeconomic losses in Japan: A cohort study

**DOI:** 10.1016/j.ijregi.2024.100495

**Published:** 2024-11-20

**Authors:** Shunichiro Konishi, Katsunori Masaki, Kyoko Shimamoto, Yoko Ibuka, Rei Goto, Ho Namkoong, Shotaro Chubachi, Hideki Terai, Takanori Asakura, Jun Miyata, Shuhei Azekawa, Kensuke Nakagawara, Hiromu Tanaka, Atsuho Morita, Norihiro Harada, Hitoshi Sasano, Ai Nakamura, Yu Kusaka, Takehiko Ohba, Yasushi Nakano, Kazumi Nishio, Yukiko Nakajima, Shoji Suzuki, Shuichi Yoshida, Hiroki Tateno, Koichi Fukunaga

**Affiliations:** 1 Division of Pulmonary Medicine, Department of Medicine, Keio University School of Medicine, Shinjuku-ku, Japan; 2 Keio Global Research Institute, Tokyo, Japan; 3 Keio University Faculty of Economics, Tokyo, Japan; 4 Keio University Graduate School of Business Administration, Tokyo, Japan; 5 Department of Infectious Diseases, Keio University School of Medicine, Tokyo, Japan; 6 Department of Respiratory Medicine, Juntendo University Faculty of Medicine and Graduate School of Medicine, Tokyo, Japan; 7 Ome Municipal General Hospital, Ome, Japan; 8 Department of Pulmonary Medicine, Kawasaki Municipal Ida Hospital, Kanagawa, Japan; 9 Department of Infectious Disease, Kawasaki Municipal Ida Hospital, Kanagawa, Japan; 10 Department of Pulmonary Medicine, Saitama City Hospital, Saitama, Japan

**Keywords:** Long COVID, Labor productivity, Economic impact, Presenteeism, Absenteeism

## Abstract

**Objectives:**

We examined shifts in labor productivity and their economic ramifications among adult patients with long COVID in Japan.

**Methods:**

A total of 396 patients were categorized into three groups based on symptom progression: non-long COVID, long COVID recovered, and long COVID persistent. Patient-reported outcomes were assessed at three time intervals: 3, 6, and 12 months after COVID-19 diagnosis. Labor productivity was gauged through presenteeism and absenteeism, measured using the World Health Organization Health and Work Performance Questionnaire.

**Results:**

Long COVID was observed in 52.7% of patients, and 29.3% of all the patients continued to experience long COVID symptoms 1 year after diagnosis. At all three time points (3, 6, and 12 months after diagnosis), the long COVID persistent group showed a statistically significant difference in absolute presenteeism compared with the non-long COVID and long COVID recovered groups (*P* <0.01). Economic loss owing to decrease in labor productivity was calculated as $21,659 per year in the long COVID persistent group and $9008 per year in the long COVID recovered group (*P* <0.01).

**Conclusion:**

The study's results revealed a notable decline in labor productivity over time, underscoring the importance of early detection and intervention to mitigate the socio-economic repercussions of long COVID, in addition to its health implications.

## Highlights

• Long COVID reduces work productivity.
• The longer long COVID lasts, the greater the amount of economic loss.
• This study assessed long COVID in Japanese society.
• Patient-reported outcomes were considered to understand the impact of long COVID.
• Results showed that symptom duration influenced lasting effects of long COVID.

## Introduction

As of January 2024, a cumulative total of 770 million individuals worldwide had been affected by COVID-19, with 7 million fatalities [1]. Although acute symptoms typically wane over time, certain symptoms may persist for several months after illness in some instances, which is termed “post-acute sequelae of SARS-CoV-2 infection” or “long COVID.” The World Health Organization [1] defines long COVID as a collection of “symptoms that persist for at least 2 months, typically 3 months or longer, subsequent to COVID-19 occurrence, with the exclusion of other diseases.”

Although the exact number of patients with long COVID is unknown, Global Burden of Disease Long COVID Collaborators et al. [2] conducted a large meta-analysis of reports from 54 countries and found that, between March 2020 and January 2022, it was present in 6.2% of all affected patients, of whom 15.1% exhibited at least one of the symptoms a year after diagnosis.

Numerous patients return to work with symptoms, which are suspected to reduce productivity in the workplace and have a significant economic and social impact. Presenteeism and absenteeism are two concepts that focus on the health of individual employees as a method of evaluating their productivity within the workplace [3]. In the Netherlands, Vaes et al. [4] utilized longitudinal data on presenteeism and absenteeism and found that 73% of patients with long COVID had been absent from work and 66% had missed work hours because of their symptoms.

Attempts have been made to quantitatively assess the impact of COVID-19 on labor productivity, most of which have been cross-sectional studies. In the United States, longitudinal data showed that long COVID resulted in reduced work hours, absenteeism, and even job turnover among patients, with a potential loss of 1 million workers and over US$ 50 billion in income per year [5]. The economic burden of COVID-19 was lower in patients with an early diagnosis compared with those with a delayed diagnosis [6]. Studies from Iran have also observed similar losses, particularly, owing to absenteeism [7,8], and loss of work hours and productivity (measured as the difference between pre- vs post–COVID-19 infection) have been investigated in the United Kingdom [9].

However, to the best of our knowledge, no reports from Asia have quantitatively analyzed the effect of long COVID on labor productivity. Furthermore, because most of the existing evidence on long COVID and labor productivity has been generated by cross-sectional studies, longitudinal studies are needed to critically examine shifting symptoms over time and the associated work productivity loss. Using data from a multicenter cohort, this study investigated the impact of long COVID on labor productivity, including absenteeism and presenteeism, and the resulting socioeconomic consequences in Japan according to socioeconomic group. It was hypothesized that labor productivity would decline further if long COVID symptoms persist.

## Methods

### World Health Organization health and work performance questionnaire

This study used the World Health Organization Health and Work Performance Questionnaire (WHO-HPQ), focusing on presenteeism and absenteeism as means for assessing labor productivity. The WHO-HPQ is a modification undertaken by the World Health Organization of the methodology published by Kessler et al*.* [10]. Validated in 2013, the Japanese version of the WHO-HPQ is publicly accessible [11]. In the WHO-HPQ, presenteeism encompasses absolute and relative measures, conceptualized as a measure of actual performance in relation to possible performance over the past 4 weeks. Absolute presenteeism measures an individual's performance, whereas relative presenteeism compares an individual's performance to the performance of others in the same or similar jobs. The same methodology is used for absenteeism: absolute absenteeism is calculated as the absenteeism (in hours) over the past 4 weeks, whereas relative absenteeism quantifies the ratio of absenteeism to anticipated work hours [10].

### Calculating presenteeism and absenteeism

The questions were used in the questionnaire to measure absolute and relative presenteeism and absenteeism (Supplementary Table 1).

### Data collection

Adult patients who were diagnosed with COVID-19 by SARS-CoV-2 polymerase chain reaction, hospitalized between February 2020 and February 2021 at 26 Japan COVID-19 Task Force-supported cooperating hospitals and who agreed to cooperate were included in the study. Patient-reported outcomes were obtained at 3, 6, and 12 months after COVID-19 diagnosis, either by a paper questionnaire sent by mail (paper patient-reported outcome) or as electronic information through a smartphone app (electronic patient-reported outcome). Medical information was collected through an electronic data capture system [12]. The respondents’ social background (Table 1) as of January 2020 was recorded. Respondents also reported the category of their annual income in 2019 (Supplementary Table 2). We used the average exchange rate in February 2022, $1 = 115.21 Japanese yen, to convert the respondents’ income to US dollars. A total of 25 symptoms were selected for this study (Table 2). The presence of any one of these symptoms was considered indicative of long COVID.

**Table 1:** Patient characteristics.

	Non-long COVID (n = 187)	Long COVID recovered (n = 91)	Long COVID persistent (n = 118)	*P-*value
Age, mean (95% CI)	52 (50.1-54.4)	52 (49.6-55.2)	52 (49.7-55.2)	1.00
Male, n (%)	131 (70)	55 (60)	64 (54)	0.02
Oxygen demand during admission, n (%)	47 (25)	25 (27)	31 (26)	1.00
Body mass index, mean (95% CI)	24 (23.8-24.9)	25 (23.9-25.2)	25 (23.6-25.3)	1.00
History of smoking, n (%)	71 (38)	35 (39)	59 (50)	0.19
Healthcare worker, n (%)	26 (14)	9 (10)	13 (11)	1.00
Annual income (USD), mean (95% CI)	47,971 (42,365-53,577)	50,123 (41,038-59,209)	41,634 (35,666-47,601)	0.26
Living with spouse, n (%)	112 (60)	76 (78)	66 (56)	0.003
University graduate or above, n (%)	95 (51)	47 (52)	49 (38)	0.10
Job, n (%)	168 (90)	89 (98)	109 (92)	0.08
Regular employment, n (%)	133 (71)	65 (71)	84 (71)	1.00
Number of hospital visits per month, mean (95% CI)	0.4 (0.25-0.55)	0.3 (0.17-0.49)	1.0 (0.70-1.22)	<0.001
Number of caregivers, mean (95% CI)	0.1 (0.05-0.14)	0.1 (0.04-0.16)	0.15 (0.07-0.22)	0.68
Number of children, mean (95% CI)	0.3 (0.15-0.36)	0.5 (0.25-0.65)	0.3 (0.24-0.40)	0.16

CI, confidence interval; USD, US dollars.

**Table 2:** Relationship between long COVID symptoms and work performance.

(a) Relationship between long COVID symptoms and presenteeism.									
Symptom	Absolute presenteeism (3 months)		*P*-value	Absolute presenteeism (6 months)		*P*-value	Absolute presenteeism (12 months)		*P*-value
	Yes	No		Yes	No		Yes	No	
Fever (≥37°C)	49 (25.3)	62 (24.6)	0.05	58 (24.4)	63 (24.0)	0.54	49 (25.3)	62 (24.6)	0.67
Cough	54 (26.0)	62 (24.5)	0.08	51 (29.4)	64 (23.5)	0.06	54 (26.0)	62 (24.5)	0.03
Phlegm	48 (24.4)	62 (24.5)	0.01	60 (25.3)	63 (24.0)	0.59	48 (24.4)	62 (24.5)	0.76
Shortness of breath	55 (26.8)	63 (24.2)	0.05	53 (28.8)	64 (23.3)	0.03	55 (26.8)	63 (24.2)	0.006
Sound, light, scent	57 (33.0)	62 (24.5)	0.68	47 (37.7)	63 (23.5)	0.19	57 (33.0)	62 (24.5)	0.11
Fatigue	48 (26.7)	65 (22.9)	<0.001	44 (27.1)	66 (22.0)	<0.001	48 (26.7)	65 (22.9)	<0.001
Hair loss	52 (27.9)	63 (24.0)	0.01	57 (28.8)	64 (23.5)	0.17	52 (27.9)	63 (24.0)	0.18
Arthritis	44 (30.3)	63 (24.0)	0.01	47 (30.5)	64 (23.1)	0.008	44 (30.3)	63 (24.0)	0.009
Muscle pain	46 (25.3)	62 (24.4)	0.005	46 (30.0)	64 (23.3)	0.013	46 (25.3)	62 (24.4)	0.04
Loss of strength	52 (26.8)	63 (24.2)	0.01	44 (29.5)	64 (23.0)	0.001	52 (26.8)	63 (24.2)	0.005
Headache	46 (27.6)	63 (23.9)	<0.001	49 (25.9)	64 (23.6)	0.006	46 (27.6)	63 (23.9)	0.015
Sore throat	52 (24.1)	62 (24.7)	0.13	70 (23.4)	63 (24.1)	0.39	52 (24.1)	62 (24.7)	0.35
Tinnitus	45 (26.0)	62 (24.5)	0.04	60 (30.2)	63 (23.9)	0.79	45 (26.0)	62 (24.5)	0.07
Impaired consciousness	57 (11.5)	61 (24.8)	0.55	65 (21.2)	63 (24.1)	0.91	57 (11.5)	61 (24.8)	0.37
Stomachache	32 (28.6)	62 (24.5)	0.08	40 (25.2)	63 (23.8)	0.05	32 (28.6)	62 (24.5)	0.36
Diarrhea	41 (27.1)	62 (24.5)	0.05	42 (28.2)	64 (23.7)	0.04	41 (27.1)	62 (24.5)	0.04
Rash of the skin	48 (29.3)	62 (24.4)	0.06	52 (30.5)	63 (23.7)	0.17	48 (29.3)	62 (24.4)	0.09
Numbness in the limbs	44 (23.4)	63 (24.4)	<0.001	47 (27.5)	64 (23.6)	0.02	44 (23.4)	63 (24.4)	0.04
Eye problem	43 (21.7)	62 (24.5)	<0.001	45 (28.0)	64 (23.6)	0.02	43 (21.7)	62 (24.5)	0.09
Memory impairment	42 (24.7)	63 (24.1)	<0.001	48 (29.5)	64 (23.3)	0.01	42 (24.7)	63 (24.1)	0.03
Brain fog	41 (29.1)	64 (22.9)	<0.001	45 (27.5)	65 (22.5)	<0.001	41 (29.1)	64 (22.9)	<0.001
Sleep disorder	42 (31.3)	64 (22.8)	<0.001	40 (27.9)	65 (22.3)	<0.001	42 (31.3)	64 (22.8)	<0.001
Taste disorder	55 (25.6)	62 (24.5)	0.07	54 (30.2)	64 (23.3)	0.09	55 (25.6)	62 (24.5)	0.17
Scent disorder	58 (25.5)	62 (24.6)	0.34	56 (25.8)	64 (23.8)	0.09	58 (25.5)	62 (24.6)	0.26
Others	61 (23.4)	61 (24.9)	0.94	59 (29.4)	63 (23.8)	0.63	61 (23.4)	61 (24.9)	0.06

(b) Relationship between long COVID symptoms and absenteeism.									
Symptom	Absolute absenteeism (3 months)		*P*-value	Absolute absenteeism (6 months)		*P*-value	Absolute absenteeism (12 months)		*P*-value
	Yes	No		Yes	No		Yes	No	
Fever (≥37°C)	19 (64.0)	5 (58.5)	0.38	−49 (80.0)	4 (54.7)	0.08	33 (74.6)	−5 (64.4)	0.32
Cough	2 (70.0)	6 (57.5)	0.76	−5 (89.3)	3 (53.2)	0.66	41 (57.1)	−6 (64.1)	0.009
Phlegm	−16 (79.0)	7 (57.0)	0.17	−25 (81.9)	4 (53.8)	0.14	−4 (53.9)	−4 (64.8)	0.98
Shortness of breath	15 (69.1)	4 (56.6)	0.27	−3 (68.9)	3 (54.4)	0.53	12 (46.8)	−6 (65.7)	0.15
Sound, light, scent	36 (84.0)	5 (57.8)	0.27	−41 (75.3)	4 (54.9)	0.01	0 (84.6)	−5 (63.8)	0.87
Fatigue	10 (67.5)	5 (56.3)	0.49	−3 (52.3)	4 (56.4)	0.40	4 (53.6)	−6 (65.8)	0.34
Hair loss	20 (72.2)	4 (56.1)	0.12	0 (57.0)	3 (55.8)	0.76	13 (53.7)	−5 (64.8)	0.29
Arthritis	17 (82.3)	5 (57.0)	0.50	0 (69.2)	3 (54.8)	0.78	2 (59.3)	−5 (64.7)	0.68
Muscle pain	29 (54.2)	4 (58.8)	0.05	−2 (79.1)	3 (54.3)	0.71	−3 (57.7)	−4 (64.7)	0.92
Loss of strength	23 (80.1)	4 (55.2)	0.07	4 (70.7)	3 (54.7)	0.90	5 (48.6)	−5 (65.3)	0.47
Headache	3 (60.0)	6 (58.7)	0.77	−14 (62.3)	4 (55.3)	0.06	−17 (63.6)	−4 (64.5)	0.37
Sore throat	−6 (78.3)	7 (57.8)	0.54	−9 (94.8)	3 (54.8)	0.53	25 (82.3)	−5 (64.1)	0.22
Tinnitus	36 (73.7)	5 (58.0)	0.17	0 (38.2)	3 (56.2)	0.90	−8 (67.9)	−4 (64.4)	0.85
Impaired consciousness	−65 (118.4)	7 (58.0)	0.40	−186 (93.3)	4 (54.1)	<0.001	−5 (73.8)	−4 (64.4)	1.00
Stomachache	34 (147.2)	6 (57.0)	0.69	26 (66.9)	2 (55.6)	0.27	43 (64.9)	−5 (64.4)	0.20
Diarrhea	−1 (93.8)	6 (57.8)	0.82	−48 (122.5)	4 (52.6)	0.22	8 (78.3)	−5 (64.3)	0.64
Rash of the skin	18 (71.6)	5 (58.1)	0.50	6 (62.7)	3 (55.7)	0.84	21 (72.8)	−5 (64.2)	0.26
Numbness in the limbs	16 (80.5)	5 (57.1)	0.54	−8 (80.8)	3 (54.4)	0.41	6 (59.2)	−5 (64.6)	0.59
Eye problem	18 (44.4)	5 (59.4)	0.25	−1 (60.9)	3 (55.7)	0.81	−4 (83.7)	−4 (64.1)	0.98
Memory impairment	1 (56.4)	6 (59.0)	0.61	4 (47.9)	3 (56.4)	0.87	0 (60.8)	−5 (64.7)	0.76
Brain fog	6 (72.2)	6 (57.0)	0.99	−15 (70.6)	5 (53.3)	0.08	7 (59.3)	−5 (64.8)	0.31
Sleep disorder	13 (64.3)	5 (58.1)	0.47	−10 (55.2)	4 (55.8)	0.16	2 (67.7)	−5 (64.3)	0.63
Taste disorder	−2 (68.0)	7 (57.4)	0.33	−3 (67.8)	3 (54.8)	0.59	−6 (56.8)	−4 (64.9)	0.93
Scent disorder	2 (62.1)	7 (58.3)	0.59	−11 (74.4)	4 (53.3)	0.10	−10 (55.3)	−4 (65.1)	0.64
Others	7 (56.6)	6 (59.0)	0.94	3 (73.3)	3 (55.2)	1.00	−9 (54.6)	−4 (64.9)	0.75

Absolute presenteeism (calculated per equation 1), mean (SD).

Absolute absenteeism (calculated per equation 3) in hours, mean (SD).

**Figure 1:**
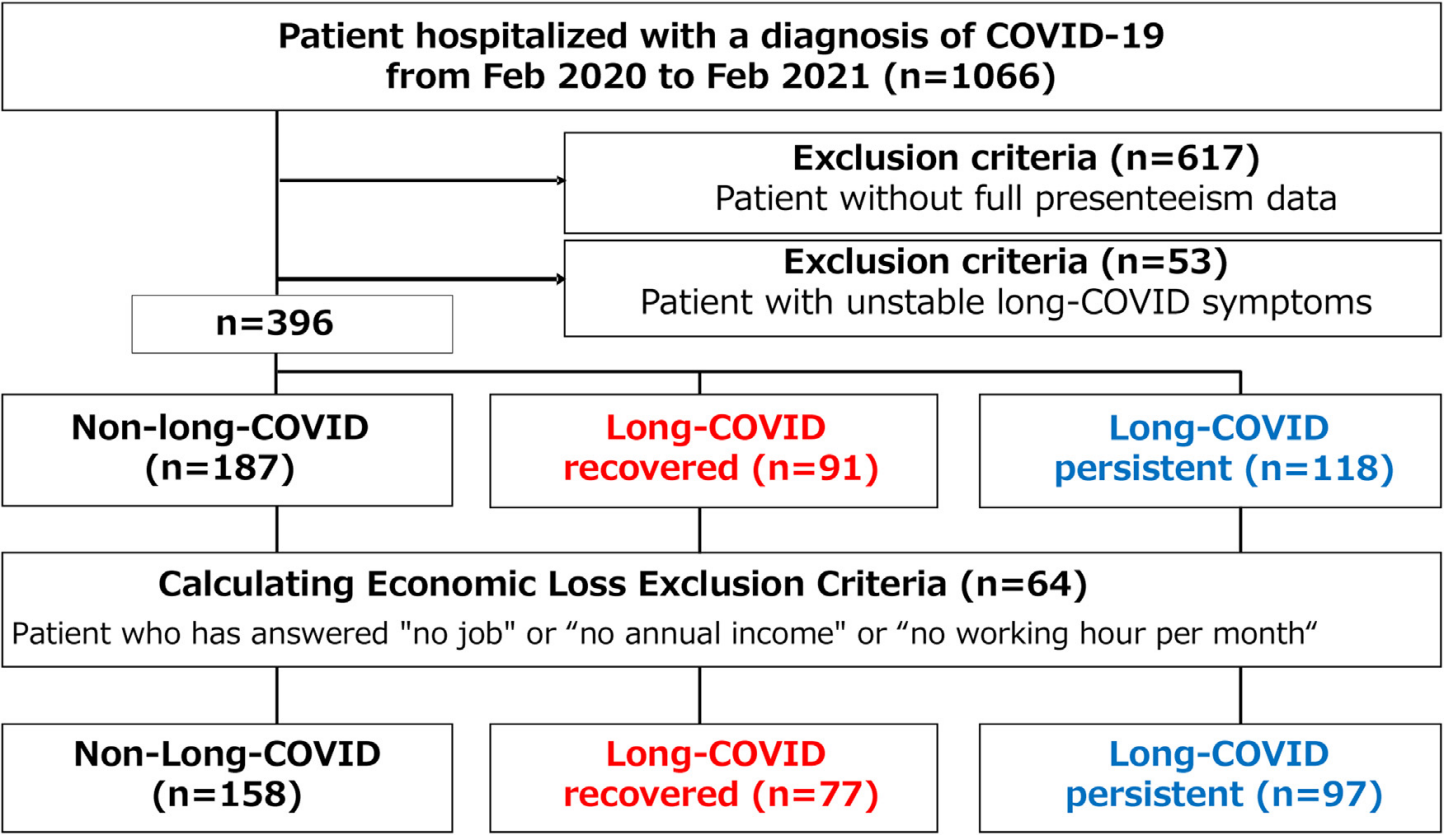
Flow diagram of participating patients.

A total of 1200 adult patients were included in the study, of whom 396 were divided into three groups—“non-long COVID”, “long COVID recovered”, and “long COVID persistent”—according to the course of their symptoms (Figure 1). The non-long COVID group (n = 187) was defined as patients with no symptoms at 3 months after diagnosis of COVID-19. The long COVID persistent group (n = 118) was defined as patients consistently symptomatic from 3 to 12 months. The long COVID recovered group (n = 91) was defined as patients who had symptoms at 3 months but were symptom-free by 12 months.

### Calculating economic loss owing to a decline in labor productivity after COVID-19 diagnosis

Economic loss due to decrease in labor productivity from the time of COVID-19 diagnosis to 12 months was calculated for 332 patients who met the study criteria (Figure 1).

### Monetary conversion method for economic losses owing to absolute presenteeism

Scholars have used different steps in this calculation: Brouwer et al. [13] calculated the lost time in terms of labor productivity and converted it to monetary terms by multiplying the lost time by the hourly wage. Nagata et al. [7] used the same method. Specifically, annual earnings are assumed to be the compensation for working “always at full performance (absolute presenteeism = 100)” and for working “the hours expected by the employer,” and time lost is defined as contractual hours worked minus time worked at full performance.

In this study, we followed the method used by Brouwer et al. [13] and Nagata et al. [7] and evaluated the monetary loss of the presenteeism of patients with long COVID. The calculation focuses on the hours worked at full performance and the hours worked during long COVID are calculated by actual working hours and patient's absolute presenteeism, respectively.

Following Brouwer et al. [13], the hours worked at full performance during long COVID were calculated by multiplying the time the respondent was originally expected to work minus the time they were absent from work by the percentage of performance loss (Supplementary Table 3).

### Method of converting the amount of economic loss owing to absolute absenteeism into monetary value

Hoeijenbos et al. [8] quantified days of sickness absence over a 3-month period and converted it into a monetary value. In the present study, absolute absenteeism was defined as hours missed per month for greater precision. Similar to absolute presenteeism, the calculation was divided into 3, 6, and 12 months after a COVID-19 diagnosis (Supplementary Table 3).

### Monetary conversion method of economic losses owing to decline in labor productivity

Because labor productivity is measured in terms of presenteeism and absenteeism, the amount of economic loss owing to the decline in labor productivity was defined as the sum of the respective loss amounts (Supplementary Table 3).

### Statistical analysis

The analysis was based on data from the three groups described previously as a backward cohort study. IBM SPSS Statistics version 28.0.1.0 (142) and GraphPad Prism 8 (GraphPad Software, San Diego, CA, USA) were utilized for all analyses and figure generation. A Student's *t*-test or one-way analysis of variance (*post hoc*: Bonferroni method) was performed, and bivariate associations were used.

## Results

### Descriptive findings

Long COVID was observed in 52.7% of all patients. The long COVID persistent group exhibited a lower proportion of males than the non-long COVID or long COVID recovered group. Furthermore, the long COVID persistent group had significantly more hospital visits per month up to 3 months after diagnosis than the other groups (Table 1). In addition, more patients in the long COVID recovered group lived with their spouses than those in the long COVID persistent group (78.0% vs 56.4%, *P* = 0.020). However, there were no disparities in age or the percentage of patients with oxygen demand during hospitalization, as has been previously reported [14].

### Long COVID and labor productivity: presenteeism and absenteeism

**Figure 2:**
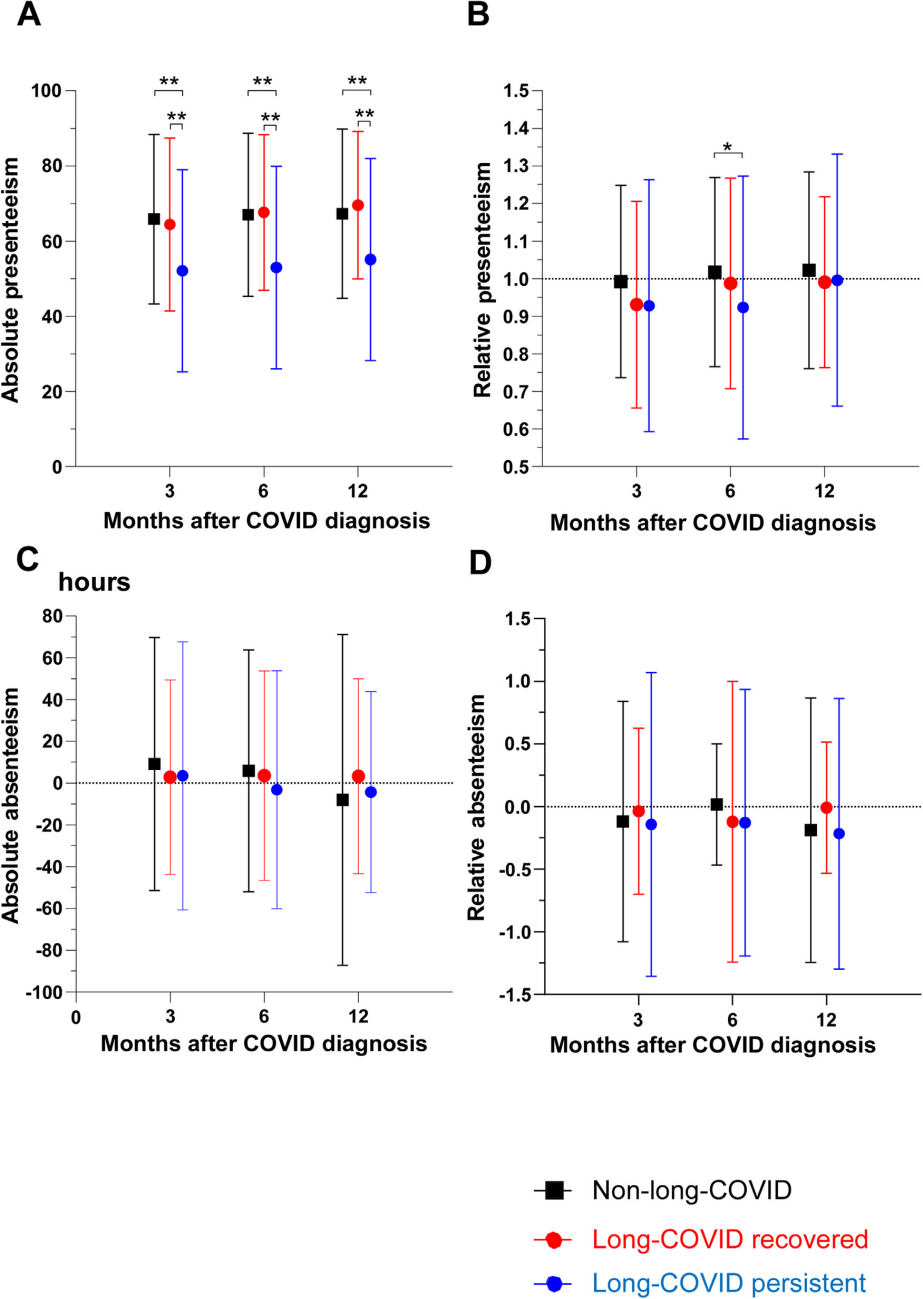
Presenteeism and absenteeism of patients after long COVID.

An analysis of absolute presenteeism (Figure 2a) demonstrated that the long COVID persistent group had significantly lower absolute presenteeism at all time points after the diagnosis of COVID-19 than the other two groups. At all three time points (3, 6, and 12 months after diagnosis), the long COVID persistent group showed a statistically significant difference in absolute presenteeism compared with the non-long COVID and long COVID recovered groups (*P* <0.01). Specifically, at 3 months, the mean values were 65.9 for non-long COVID, 64.4 for long COVID recovered, and 52.1 for long COVID persistent (*P* <0.01 for comparisons of the long COVID persistent group with the long COVID recovered and non-long COVID groups). At 6 months, the mean values were 67.0 for non-long COVID, 67.6 for long COVID recovered, and 53.0 for long COVID persistent (*P* <0.01). Similarly at 12 months, the mean values were 67.2 for non-long COVID, 69.6 for long COVID recovered, and 55.1 for long COVID persistent (*P* <0.01). The same analysis was conducted for relative presenteeism (Figure 2b), with significantly lower relative presenteeism in the long COVID persistent group than the non-long COVID group at 6 months after COVID-19 diagnosis (1.0167 vs 0.9229, *P* = 0.025).

The analysis for absolute absenteeism (Figure 2c) showed no significant disparities between the groups. Relative absenteeism was also not significantly different between groups or time points; it was negative in all groups beginning at 3 months and remained the same at 12 months (Figure 2d). All groups worked more than workers in the same office or occupation.

### Economic loss owing to decreased labor productivity while experiencing long COVID

**Figure 3:**
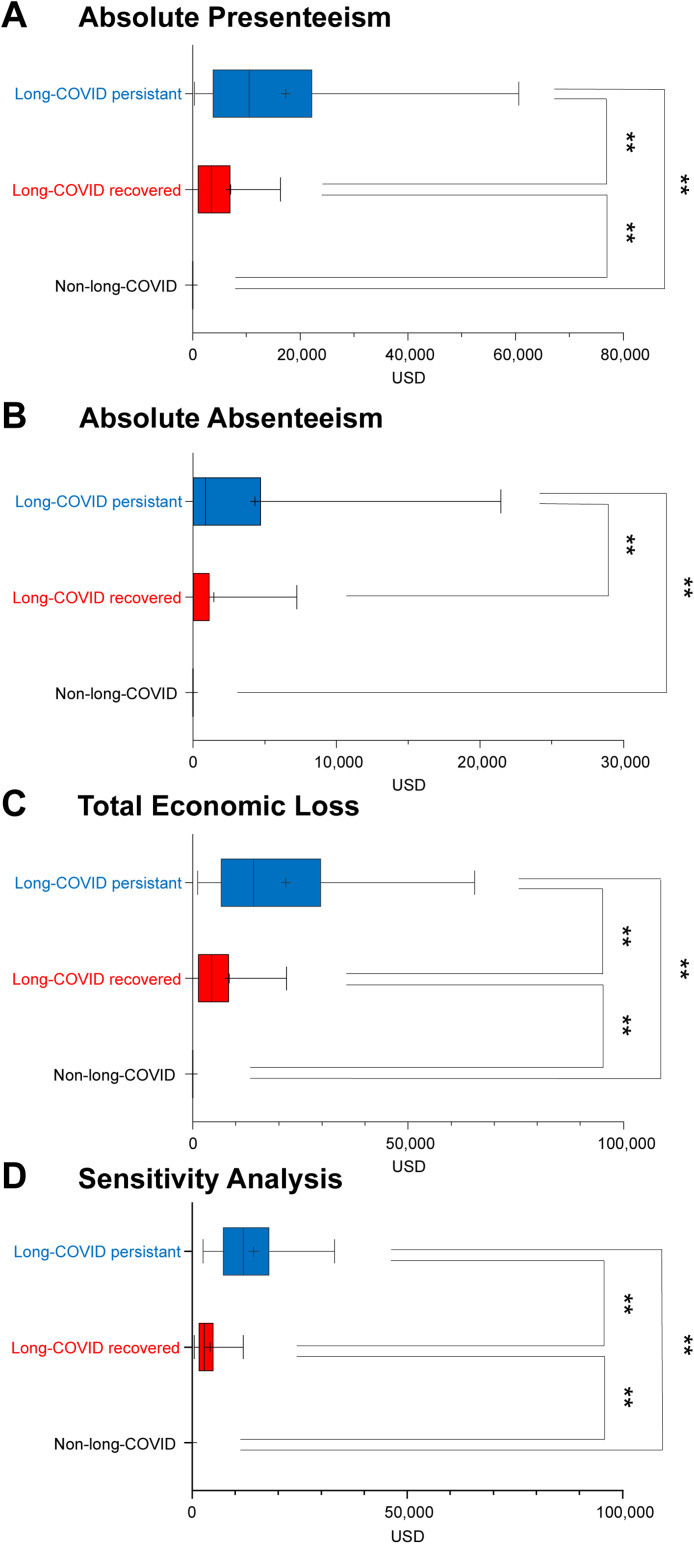
Economic loss due to the presenteeism and absenteeism of patients after long COVID.

In terms of absolute presenteeism, the long COVID persistent group exhibited an incremental economic loss of $17,334 per year (*P* <0.01) compared with the non-long COVID group and $9860 per year (*P* <0.01) compared with the long COVID recovered group. The long COVID recovered group also exhibited an economic loss of $7474 per year (*P* <0.01) compared with the non-long COVID group (Figure 3a).

Regarding the amount of economic loss because of absolute absenteeism, the median was $882 per year more in the long COVID persistent group than in the other groups. From a comparison of mean values, the long COVID persistent group exhibited an annual loss of $4325, which was more than the annual loss of $1534 in the long COVID recovered group (Figure 3b).

The economic loss owing to the decrease in labor productivity, which was the sum of absolute presenteeism and absolute absenteeism, was $21,659 per year in the long COVID persistent group and $9008 per year in the long COVID recovered group; thus, the disparity in economic loss between these two groups was $12,651 per year (*P* <0.01) (Figure 3c). We performed univariate and multivariate analyses of factors associated with differences between long COVID persistent and long COVID recovered groups (Table 1). We found that being unmarried and the number of hospital visits within 3 months after the diagnosis were significantly associated with the presence or absence of prolonged sequelae per univariate analysis. However, there was no association between employment status or economic loss due to COVID-19 and the presence of persistent sequelae. The multivariate analysis showed that the number of hospital visits per month until 3 months after diagnosis had a statistically significant association (odds ratio 1.72, 95% confidence interval 1.17-2.53) (Supplementary Table 4).

### Sensitivity analysis

To check the robustness of this estimate in consideration of the participants’ relatively high annual incomes, we substituted them with annual incomes adjusted for gender and age based on the results of the Ministry of Health, Labour and Welfare's 2020 annual income survey [15]. Total economic loss because of decreased labor productivity was $14,245 per year in the long COVID persistent group and $4226 per year in the long COVID recovered group, exhibiting a disparity of $10,019 per year (*P* <0.01) between these two groups (Figure 3d).

### Decline in labor productivity and long COVID symptoms

The relationship between long COVID symptoms and absolute presenteeism of patients at each time point since COVID-19 diagnosis is depicted in Table 2a. This table shows patients with and without long COVID symptoms at 3, 6, and 12 months after diagnosis. The mean absolute presenteeism at each time point was compared. Patients with the long COVID symptoms of fatigue, brain fog, and sleep disturbances tended to have lower absolute presenteeism at any given point. The relationship between absolute absenteeism and long COVID symptoms at each time point after COVID-19 diagnosis is presented in Table 2b. There were no symptoms for which absolute absenteeism was significantly reduced across all periods.

## Discussion

In total, 29.3% of patients had long COVID a year after diagnosis. Patients with long COVID that persisted for 1 year exhibited a significant decrease in labor productivity compared with those who did not; for these patients, the economic loss owing to diminished labor productivity was $21,659 per year. Furthermore, patients whose long COVID persisted for 1 year had a significantly higher annual loss of $12,651 than those with mid-year resolution. The sensitivity analysis also demonstrated a disparity of $10,019 per year, indicating that economic losses were greater if long COVID persisted; that is, the longer the long COVID continued, the greater the losses. The multivariate analysis indicated that the more frequently a patient visits the hospital within 3 months of being diagnosed with COVID-19, the more likely it is that their symptoms will remain (odds ratio 1.72, 95% confidence interval 1.17-2.53) (Supplementary Table 4). Symptoms such as fatigue, brain fog, and sleep disorders had lower presenteeism at all time points and may be possible risk factors that warrant further research (Table 2a).

This study's unique contribution lies in its focus on patients experiencing long COVID symptoms over a year, which revealed a notable decline in labor productivity over time. This underscores the importance of early detection and intervention to mitigate the socioeconomic repercussions of long COVID, in addition to its health implications.

A limitation of this study is that the economic loss may have been overestimated. First, during the spread of COVID-19, social factors other than COVID-19 may have influenced presenteeism; however, in this study, the presenteeism of individuals who did not have COVID-19 was not included. There are no data about intermittent, multifactorial changes of presenteeism in those groups; therefore, it is unclear if the results reflect changes in labor productivity or economic losses caused solely by long COVID. Nevertheless, this study shows that the longer the long COVID symptoms persist in patients, the lower their labor productivity and the greater the amount of loss.

Second, we only asked patients about their annual income and work as of 2019 and not about any changes after they contracted COVID-19, meaning that some patients may have lost their jobs or had a decrease in their annual income. Buonsenso et al. [16] investigated the impact of COVID-19 sequelae on employment status and found that 19.0% of patients reported not feeling fully recovered within 1 year of the acute illness, and 13.7% reported a change in their job status after COVID-19. We conducted a sensitivity analysis and found that there were significant differences in labor productivity between the long COVID recovered and long COVID persistent groups (Figure 3d). We also found that there were significant differences between the long COVID recovered and long COVID persistent groups (Figure 3d). The long COVID persistent group's average economic loss, which was calculated using annual income in our study ($21,659 per year), and that determined by the Ministry of Health, Labour and Welfare database ($14,245 per year) differed substantially ($7414), and further investigation is needed to determine the specific amounts of losses.

Third, absenteeism took into account all time off rather than only sick leave and could, therefore, have included childcare leave. However, unlike in Figure 3b, this does not recognize the amount of economic loss due to absolute absenteeism. In fact, even if it includes days off other than sick leave, this can be ignored as people are working more than expected.

Finally, this study may have been affected by selection bias because it excluded patients for whom data on presenteeism were not available, and the participating patients had relatively higher annual incomes than the average annual income in Japan.

Conversely, economic loss may have been underestimated. First, to represent an annual income, cases with incomes of 14 million yen or more were categorized as “16 million yen,” potentially leading to an underestimation for patients with incomes surpassing 14 million yen. Second, welfare benefits have been factored into studies reporting on other diseases, which results in an additional 20% being added to the annual salary in these studies [7]. However, it is important to note that this study included patients from diverse work environments, making it challenging to uniformly and accurately account for welfare benefits. Because our objective was to determine the minimum amount of economic loss resulting from persistent long COVID, we opted not to calculate welfare benefits as part of the patients’ salaries.

Furthermore, the timing of this study necessitates caution when interpreting its results. The study targeted patients from the first wave of COVID-19 in Japan, which spanned from February to May 2020, the second wave from June to October 2020, and the third wave from November 2020 to February 2021. Vaccination efforts commenced after the third wave, and it is anticipated that vaccinations and therapeutic medications will have mitigated the incidence of long COVID [17]. However, given the evolving landscape of virus variants, caution is warranted when extrapolating these findings to current patients with COVID-19.

## Conclusion

In total, 29.3% of the patient cohort continued to experience long COVID symptoms 1 year after COVID-19 diagnosis. At 3, 6, and 12 months after diagnosis, patients with long COVID that persisted for 1 year were more prevalent than those with early resolution of long COVID or those without long COVID. Presenteeism, a key labor productivity indicator, exhibited a significant decrease in these patients, suggesting an association between reduced work productivity and long COVID that persisted for 1 year. For these patients, the annual economic loss attributed to diminished labor productivity amounted to $21,659. As the duration of COVID-19 persistence increased, the associated economic losses grew significantly higher. A substantial proportion of patients experiencing long COVID symptoms, such as fatigue, impaired cognitive function, and sleep disturbances, demonstrated a marked decline in presenteeism. Quantitative evaluations of the impact of long COVID on labor productivity remain limited in Asia and worldwide, indicating a need for further research to comprehensively assess its societal implications.

## Declarations of competing interest

The authors have no competing interests to declare.

## Appendix Supplementary materials

Image, application 1

Image, application 2

Image, application 3

Image, application 4
