# Supplementary material for: The relationship between long COVID, labor productivity, and socioeconomic losses in Japan: A cohort study
Source: IJID Reg. 2024 Nov 20;14:100495. doi: 10.1016/j.ijregi.2024.100495 (PMC11664411; doi:10.1016/j.ijregi.2024.100495)
Supplement: Supplementary file 1 [file mmc1.docx]

Supplementary Table 4. Factors contributing to the persistence of long COVID—multivariable analysis

|  | **Odds ratio** | **95% CI** | **p-value** |
| --- | --- | --- | --- |
| Age, year | 1.00 | 0.97–1.03 | 0.88 |
| Male | 0.72 | 0.32–1.63 | 0.43 |
| Oxygen demand during admission | 0.89 | 0.39–2.06 | 0.79 |
| Regular employment | 1.38 | 0.56–3.39 | 0.48 |
| Living with spouse | 0.54 | 0.26–1.16 | 0.11 |
| Number of hospital visits per month, until 3 months after the diagnosis | 1.72 | 1.17–2.53 | 0.006 |
| Economic loss, USD | 1.00 | 1.00–1.00 | 0.32 |

*Note.* COVID, coronavirus disease; CI, confidence interval; USD, United States dollar
