# Supplementary material for: The relationship between long COVID, labor productivity, and socioeconomic losses in Japan: A cohort study
Source: IJID Reg. 2024 Nov 20;14:100495. doi: 10.1016/j.ijregi.2024.100495 (PMC11664411; doi:10.1016/j.ijregi.2024.100495)
Supplement: Supplementary file 2 [file mmc2.pptx]

## Slide 1
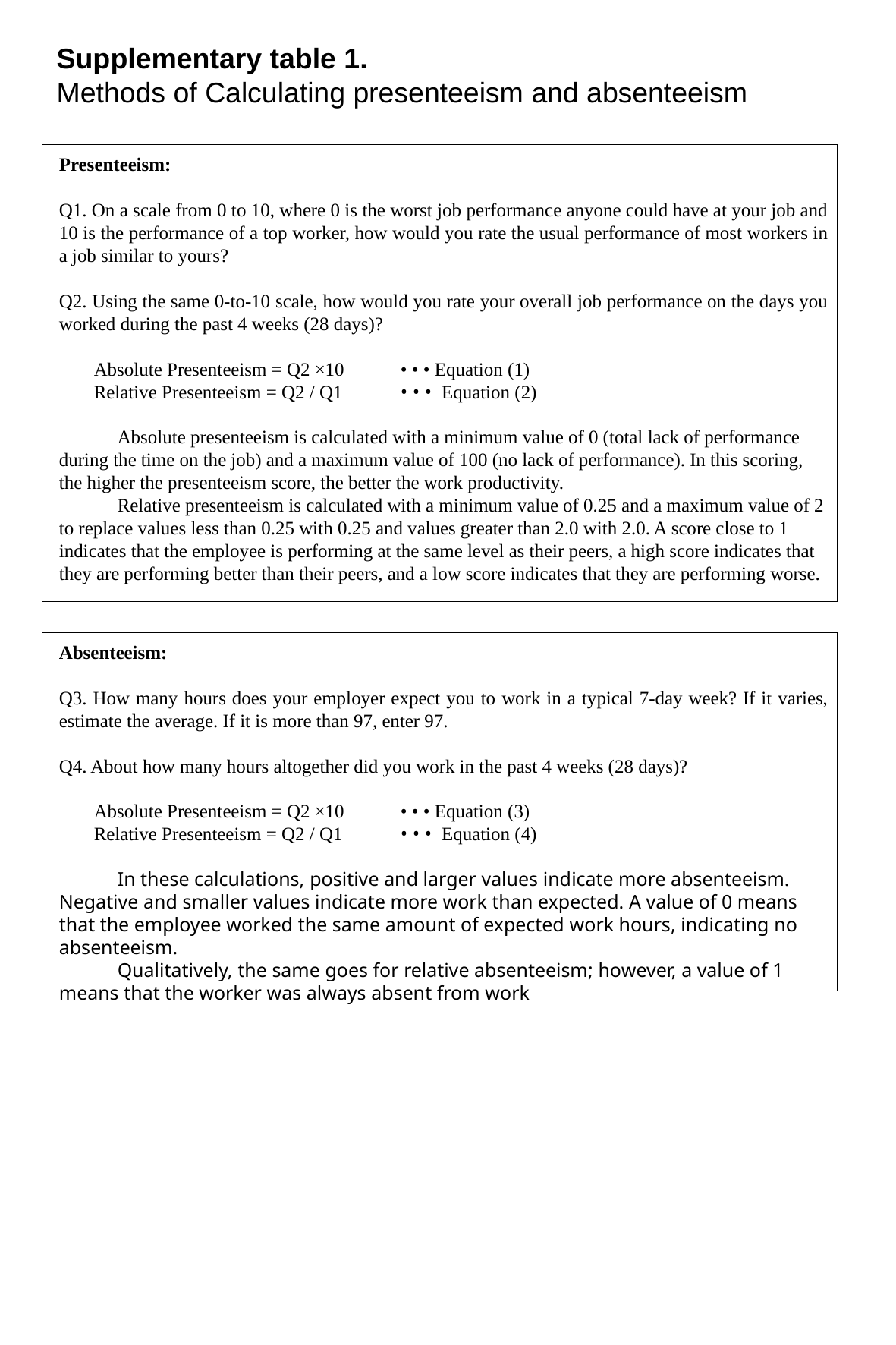

Supplementary table 1.
Methods of Calculating presenteeism and absenteeism
Presenteeism:
Q1. On a scale from 0 to 10, where 0 is the worst job performance anyone could have at your job and 10 is the performance of a top worker, how would you rate the usual performance of most workers in a job similar to yours?
Q2. Using the same 0-to-10 scale, how would you rate your overall job performance on the days you worked during the past 4 weeks (28 days)?
Absolute Presenteeism = Q2 ×10	• • • Equation (1)
Relative Presenteeism = Q2 / Q1　 	• • • Equation (2)
　Absolute presenteeism is calculated with a minimum value of 0 (total lack of performance during the time on the job) and a maximum value of 100 (no lack of performance). In this scoring, the higher the presenteeism score, the better the work productivity.
　Relative presenteeism is calculated with a minimum value of 0.25 and a maximum value of 2 to replace values less than 0.25 with 0.25 and values greater than 2.0 with 2.0. A score close to 1 indicates that the employee is performing at the same level as their peers, a high score indicates that they are performing better than their peers, and a low score indicates that they are performing worse.
Absenteeism:
Q3. How many hours does your employer expect you to work in a typical 7-day week? If it varies, estimate the average. If it is more than 97, enter 97.
Q4. About how many hours altogether did you work in the past 4 weeks (28 days)?
Absolute Presenteeism = Q2 ×10	• • • Equation (3)
Relative Presenteeism = Q2 / Q1　　	• • • Equation (4)
　In these calculations, positive and larger values indicate more absenteeism. Negative and smaller values indicate more work than expected. A value of 0 means that the employee worked the same amount of expected work hours, indicating no absenteeism.
　Qualitatively, the same goes for relative absenteeism; however, a value of 1 means that the worker was always absent from work
