# Supplementary material for: The relationship between long COVID, labor productivity, and socioeconomic losses in Japan: A cohort study
Source: IJID Reg. 2024 Nov 20;14:100495. doi: 10.1016/j.ijregi.2024.100495 (PMC11664411; doi:10.1016/j.ijregi.2024.100495)
Supplement: Supplementary file 3 [file mmc3.pptx]

## Slide 1
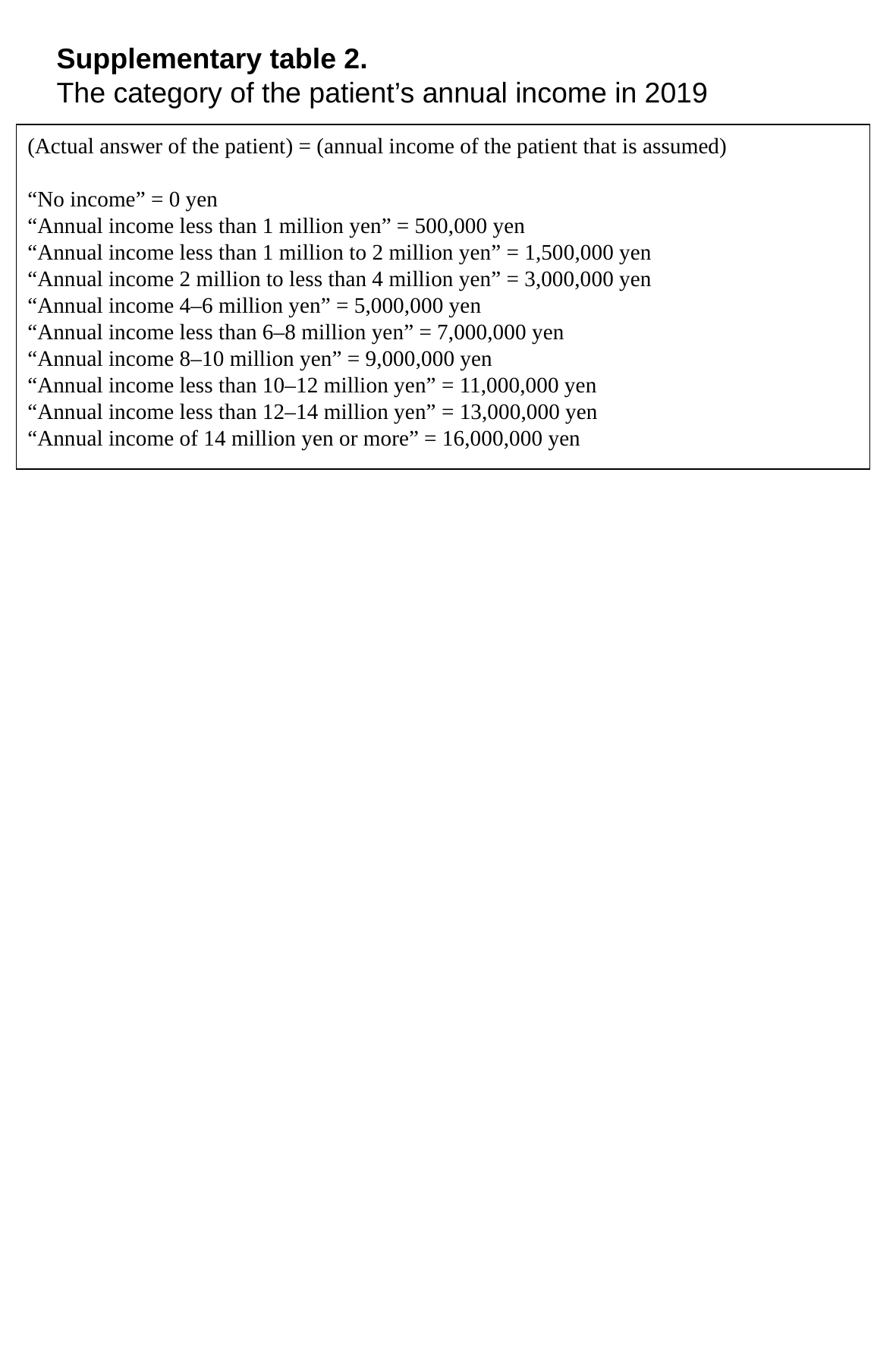

Supplementary table 2.
The category of the patient’s annual income in 2019
(Actual answer of the patient) = (annual income of the patient that is assumed)
“No income” = 0 yen
“Annual income less than 1 million yen” = 500,000 yen
“Annual income less than 1 million to 2 million yen” = 1,500,000 yen
“Annual income 2 million to less than 4 million yen” = 3,000,000 yen
“Annual income 4–6 million yen” = 5,000,000 yen
“Annual income less than 6–8 million yen” = 7,000,000 yen
“Annual income 8–10 million yen” = 9,000,000 yen
“Annual income less than 10–12 million yen” = 11,000,000 yen
“Annual income less than 12–14 million yen” = 13,000,000 yen
“Annual income of 14 million yen or more” = 16,000,000 yen
