# Supplementary material for: The relationship between long COVID, labor productivity, and socioeconomic losses in Japan: A cohort study
Source: IJID Reg. 2024 Nov 20;14:100495. doi: 10.1016/j.ijregi.2024.100495 (PMC11664411; doi:10.1016/j.ijregi.2024.100495)
Supplement: Supplementary file 4 [file mmc4.pptx]

## Slide 1
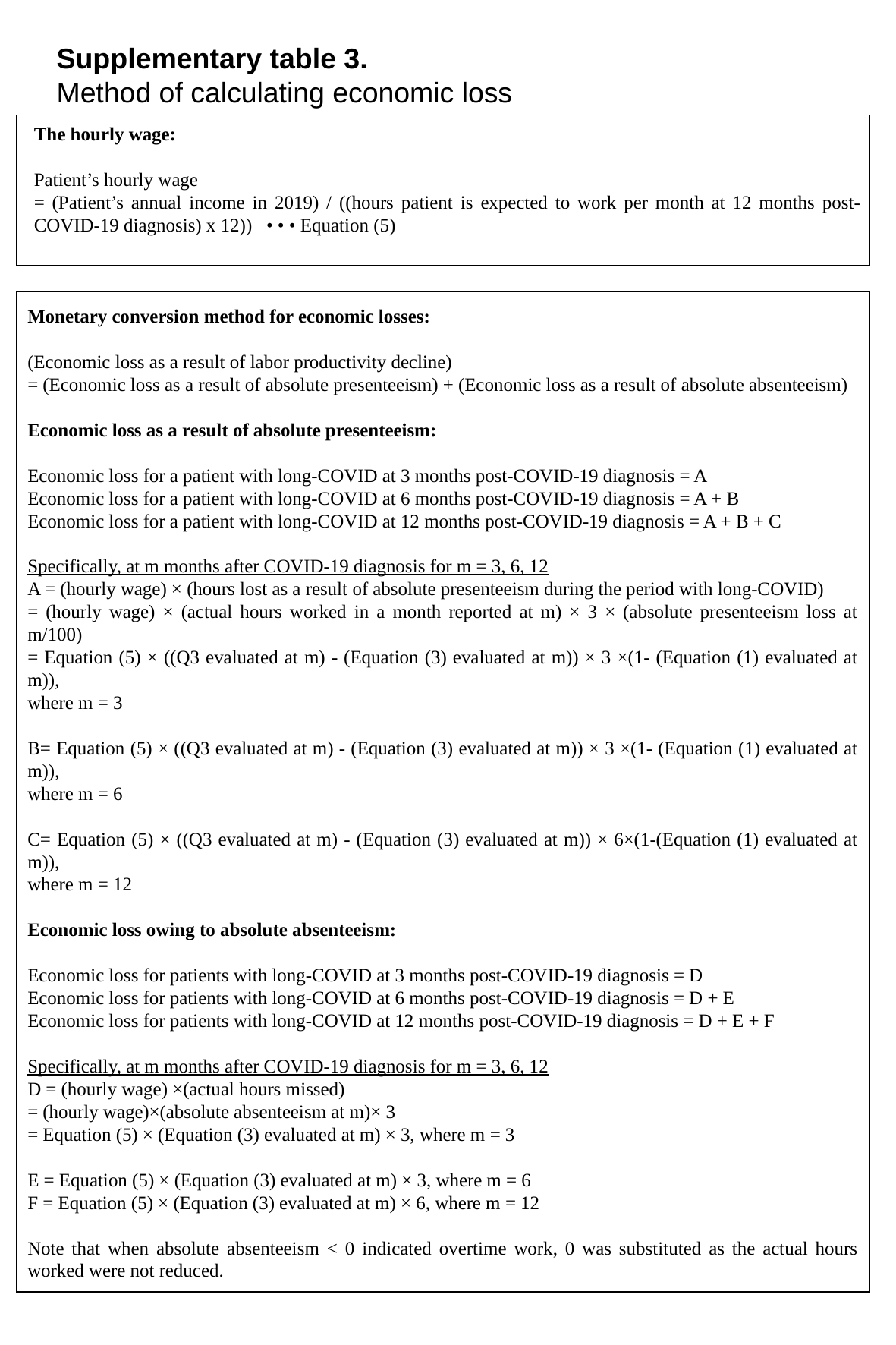

Supplementary table 3.
Method of calculating economic loss
The hourly wage:
Patient’s hourly wage
= (Patient’s annual income in 2019) / ((hours patient is expected to work per month at 12 months post-COVID-19 diagnosis) x 12))	 • • • Equation (5)
Monetary conversion method for economic losses:
(Economic loss as a result of labor productivity decline)
= (Economic loss as a result of absolute presenteeism) + (Economic loss as a result of absolute absenteeism)
Economic loss as a result of absolute presenteeism:
Economic loss for a patient with long-COVID at 3 months post-COVID-19 diagnosis = A
Economic loss for a patient with long-COVID at 6 months post-COVID-19 diagnosis = A + B
Economic loss for a patient with long-COVID at 12 months post-COVID-19 diagnosis = A + B + C
Specifically, at m months after COVID-19 diagnosis for m = 3, 6, 12
A = (hourly wage) × (hours lost as a result of absolute presenteeism during the period with long-COVID)
= (hourly wage) × (actual hours worked in a month reported at m) × 3 × (absolute presenteeism loss at m/100)
= Equation (5) × ((Q3 evaluated at m) - (Equation (3) evaluated at m)) × 3 ×(1- (Equation (1) evaluated at m)),
where m = 3
B= Equation (5) × ((Q3 evaluated at m) - (Equation (3) evaluated at m)) × 3 ×(1- (Equation (1) evaluated at m)),
where m = 6
C= Equation (5) × ((Q3 evaluated at m) - (Equation (3) evaluated at m)) × 6×(1-(Equation (1) evaluated at m)),
where m = 12
Economic loss owing to absolute absenteeism:
Economic loss for patients with long-COVID at 3 months post-COVID-19 diagnosis = D
Economic loss for patients with long-COVID at 6 months post-COVID-19 diagnosis = D + E
Economic loss for patients with long-COVID at 12 months post-COVID-19 diagnosis = D + E + F
Specifically, at m months after COVID-19 diagnosis for m = 3, 6, 12
D = (hourly wage) ×(actual hours missed)
= (hourly wage)×(absolute absenteeism at m)× 3
= Equation (5) × (Equation (3) evaluated at m) × 3, where m = 3
E = Equation (5) × (Equation (3) evaluated at m) × 3, where m = 6
F = Equation (5) × (Equation (3) evaluated at m) × 6, where m = 12
Note that when absolute absenteeism < 0 indicated overtime work, 0 was substituted as the actual hours worked were not reduced.
